# Production of transglutaminase in glutathione-producing recombinant *Saccharomyces cerevisiae*

**DOI:** 10.1186/s13568-020-01176-3

**Published:** 2021-01-07

**Authors:** Yoko Hirono-Hara, Miyuu Yui, Kiyotaka Y. Hara

**Affiliations:** 1 Department of Environmental and Life Sciences, School of Food and Nutritional Sciences, University of Shizuoka, 52-1 Yada, Suruga-ku, Shizuoka, 422-8526 Japan; 2 Graduate Division of Nutritional and Environmental Sciences, University of Shizuoka, 52-1 Yada, Suruga-ku, Shizuoka, 422-8526 Japan

**Keywords:** Transglutaminase, Glutathione, *Streptoverticillium mobaraense*, Yeast, *Saccharomyces cerevisiae*, Coproduction

## Abstract

Transglutaminase (TG) catalyzes the formation of cross-links between proteins. TG from *Streptoverticillium mobaraense* (*Sm*TG) is used widely in food, cosmetic, biomaterial and medical industries. *Sm*TG is occasionally supplied as a mixture with the activator peptide glutathione. Currently, glutathione is industrially produced using a budding yeast, *Saccharomyces cerevisiae*, because of its intracellular high content of glutathione. In this study, active *Sm*TG was produced together with glutathione in *S. cerevisiae*. *Sm*TG extracted from *S. cerevisiae* expressing *Sm*TG showed cross-linking activity when BSA and sodium caseinate were substrates. The cross-linking activity of *Sm*TG increased proportionally as the concentration of added glutathione increased. Furthermore, *Sm*TG was prepared by extracting *Sm*TG from an engineered *S. cerevisiae* whose glutathione synthetic pathway was enhanced. The *Sm*TG solution showed higher activity when compared with a *Sm*TG solution prepared from a *S. cerevisiae* strain without enhanced glutathione production. This result indicates that a high content of intracellular glutathione further enhances active *Sm*TG production in *S. cerevisiae*. *S. cerevisiae* co-producing *Sm*TG and a higher content of glutathione has the potential to supply a ready-to-use industrial active TG solution.
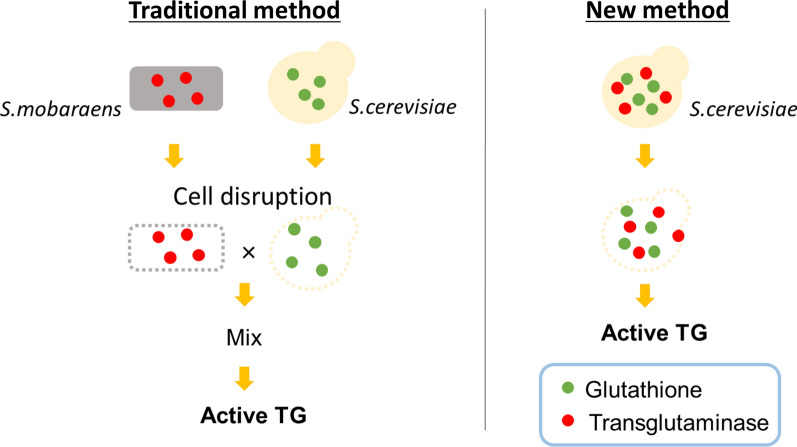

## Key points

- *Streptoverticillium mobaraense* transglutaminase (*Sm*TG) is used widely in the food industry.
- *Sm*TG is activated by the peptide glutathione.
- Active *Sm*TG was produced in an engineered high glutathione-producing *S. cerevisiae*.

## Introduction

Transglutaminase (TG; EC 2.3.2.13) is an acyl transferase that forms both intra- and inter-molecular isopeptide bonds in and between proteins. TG catalyzes an acyl transfer reaction between the γ-carboxyamide group of glutamine residues and the γ-amino group of lysine residues to form ε-(γ-glutamyl)lysine isopeptide bonds. Such reactions facilitate the polymerization of proteins (Folk 1980; Lorand and Conrad 1984; Kashiwagi et al. 2002).

TG is used widely in the food, chemical, medical and cosmetic industries because of its broad cross-linking activity (Ikura et al. 1981; Kurth and Rogers 1984; Savoca et al. 2018). In particular, TG is often used widely in the food industry to avoid chemical modification processes to link proteins (Kuraishi et al. 1997; Gerrard et al. 1998; Abou-Soliman et al. 2017), which often give rise to unwanted side reactions and require high-pressure/temperature conditions and chemical solvents (Miwa 2020). TG is distributed widely in nature and has been identified in bacteria and mammals. However, when focusing on the industrial use of TG, microbial transglutaminase produced by *S. mobaraense* (*Sm*TG) is a major choice because of various advantages that include Ca^2+^ independence, higher reaction rates, thermostability, wider substrate specificity for acyl donors and a smaller molecular size when compared with other TGs (Ando et al. 1989; Nonaka et al. 1989; Kanaji et al. 1993; Miwa 2020).

The addition of glutathione has been reported to activate *Sm*TG (Bönisch et al. 2007; Miwa 2020). Glutathione is an anti-oxidant tripeptide that is distributed widely among animals, plants and microorganisms, playing an important role in the protection of cells against oxidation and the maintenance of enzymes and other cellular components in the reduced state (Schmacht et al. 2017). Currently, industrial production of glutathione uses the budding yeast, *Saccharomyces cerevisiae*, because of its high intracellular glutathione content (Li et al. 2004). *Sm*TG mixed with yeast extract containing glutathione is commercially available (Bönisch et al. 2007; Miwa 2020). The mechanism of glutathione activating *Sm*TG remains unresolved, although Bönisch et al. (2007) demonstrated that reduction of an unknown inhibitor(s) present in milk by glutathione improved crosslinking of casein (Bönisch et al. 2007). Therefore, production of both *Sm*TG and glutathione using *S. cerevisiae* expressing *Sm*TG should be a promising, simple one-pot process to obtain highly active *Sm*TG. In *S. cerevisiae*, glutathione biosynthesis is continuously catalyzed by glutathione synthase I (Gsh1) and glutathione synthase II (Gsh2) from three precursor amino acids (Li et al. 2004; Schmacht et al. 2017). We previously developed an engineered *S. cerevisiae* strain (GCI strain) whose glutathione biosynthesis was improved by overexpression of Gsh1 and Gsh2 (Hara et al. 2012).

Several studies have reported heterologous expression of *Sm*TG in microorganisms such as *Escherichia coli* (Salis et al. 2015), *Corynebacterium glutamicum* (Itaya and Kikuchi 2008) and *Pichia pastoris* (Özçelik et al. 2019), whereas the glutathione-producing strain *S. cerevisiae* has never been used as a host strain. In the current study, *S. cerevisiae* was evaluated as a host strain to produce *Sm*TG. Moreover, the produced *Sm*TG solution was active when expressed in the engineered *S. cerevisiae* GCI strain. The results demonstrate the potential of *S. cerevisiae* as a host strain to produce industrial levels of active *Sm*TG.

## Materials and methods

### Strain and media

*Escherichia coli* DH5α cells were used for recombinant DNA manipulation. *S. cerevisiae* BY4741 strain [MATα *his3Δ*1 *ura3Δ*0 *leu2Δ*0 *met15Δ*0] and the previously engineered glutathione-hyper producing yeast (GCI strain) (Hara et al. 2015) that overexpresses the *GSH1* and *GSH2* genes were used.

*Escherichia coli* transformants were grown in Luria–Bertani medium (10 g/L tryptone, 5 g/L yeast extract, 5 g/L NaCl) supplemented with 100 µg/mL ampicillin. Yeast transformants were grown in rich yeast-extract peptone dextrose (YPD) medium (10 g/L yeast extract, 20 g/L bacto-peptone, 20 g/L glucose) supplemented with or without 0.5 µg/mL aureobasidin A (Takara Bio, Shiga, Japan), and in selective synthetic dextrose (SD) medium (6.7 g/L yeast nitrogen base without amino acids, 20 g/L glucose) supplemented with appropriate concentrations of amino acids and nucleobases.

### Plasmid and yeast transformation

The gene encoding TG from *S. mobaraense* (*Sm*TG) is deposited in the Gene bank with accession No. AF531437.1. The nucleotide sequence encoding *Sm*TG was codon optimized for production in *S. cerevisiae* (*Sm*TG; DDBJ accession No. LC583746) by Eurofins genomics (Tokyo, Japan). The synthesized DNA encoding *Sm*TG was digested with *Sal*I/*Bgl*II and inserted into the *Sal*I/*Bgl*II sites of pGK425 (Ishii et al. 2009) to give the expression vector pGK425-TG. The *S. cerevisiae* BY4741 strain and GCI strain were transformed with pGK425-TG using a lithium acetate method (Ito et al. 1983; Gietz et al. 1992) to yield the BY4741-TG and GCI-TG strains, respectively.

### Preparation of *S. mobaraense* transglutaminase

Yeast transformants were pre-cultured in 5 mL of YPD at 30 °C and 200 rpm overnight. This pre-culture was inoculated into 20 mL of SD medium in the presence of appropriate concentrations of amino acids and nucleobases for the BY4741-TG strain and additionally 0.5 µg/mL aureobasidin A for the GCI-TG strain. The inoculation volumes were adjusted to an initial OD_600_ of 0.15 in 200 mL baffled Erlenmeyer flasks and cultured at 200 rpm in a shaking incubator (BR-43FL, TAITEC, Saitama, Japan) at 30 °C.

For preparation of the *Y*×*Sm*TG (*Sm*TG extracted from *S. cerevisiae* expressing *Sm*TG) solution, the supernatant from *Y* mL of a 24 h cell culture was discarded after centrifugation at 3000×*g* for 5 min at 4 °C. The collected cells were suspended in 300 µL ice-cold TE buffer (100 mM Tris–HCl (pH 8.0), 2 mM ethylenediaminetetraacetic acid) and disrupted by a cell disruptor (Shake Master NEO, Bio Medical Sciences, Tokyo, Japan) with zirconia beads at 1500 rpm for 10 min. The cell debris were removed by centrifugation at 16,000×*g* for 10 min at 4 °C. The supernatants (*Sm*TG solution) were stored at − 80 °C until use.

### Cross-linking assay of *Sm*TG

For the BSA cross-linking assay of *Sm*TG at different reaction times, 40 µL of the reaction buffer BD [1.25 mg/mL BSA, 50 µM dithiothreitol (DTT)] and 10 µL of the 1×*Sm*TG solution in 100 mM Tris–HCl (pH 8.0) were mixed and incubated for 0, 30, 60 and 120 min at 50 °C. For the BSA cross-linking assay with different volumes of *Sm*TG solutions, 40 µL of the reaction buffer BD and 10 µL of 1×, 5×, or 10×*Sm*TG solution in 100 mM Tris–HCl (pH 8.0) were mixed and incubated for 120 min at 50 °C.

Because BSA required DTT as a reducing agent for cross-lining, the caseinate was used for cross-linking assay to evaluate the presence of several concentrations of glutathione as the other reducing agent for unknown inhibitor(s) (Bönisch et al. 2007). One hundred and sixty microliters of the reaction buffer containing 0.625 mg/mL sodium caseinate with 0–3.2 mM glutathione and 40 µL of the 1×*Sm*TG solution prepared from the BY4741-TG strain were mixed and incubated overnight at 50 °C. Forty microliters of the reaction mixture was used for sodium dodecyl sulfate-polyacrylamide gel electrophoresis (SDS-PAGE) analysis. Sixty microliters of the reaction mixture was ultra-filtered by using Amicon ultrafiltration units (Merck KGaA, Darmstadt, Germany) to remove proteins whose molecular weights were under 100 kDa, and the concentrations of the cross-linked higher molecular weight proteins (> 100 kDa) were measured by staining with Coomassie Brilliant Blue (CBB) solution (Nacalai-Tesque, Kyoto, Japan).

Cross-linking activity of *Sm*TG extracted from BY4741-TG and GCI-TG strains was measured by using caseinate as the substrate. Forty microliters of 0.625 mg/mL caseinate and 10 µL of 12×*Sm*TG solution in 100 mM Tris–HCl (pH 8.0) were mixed and incubated overnight at 50 °C. After the cross-linking reaction, 10 µL of the reaction mixture was suspended into an equal volume of sample buffer for SDS-PAGE (Nacalai-Tesque) analysis, incubated for 3 min at 100 °C, loaded onto a 12% SDS-PAGE gel, and the gel was stained by CBB stain One Super (Nacalai-Tesque). Relative level of protein was calculated from the intensity of each protein band using Image J software (https://imagej.nih.gov/ij/).

### Measurement of *Sm*TG activity

Activity of *Sm*TGs was measured by incorporation of the fluorescent amine, dansyl cadaverine [*N*-(5-aminopentyl)-5-dimethylamino-1-naphthalenesulfonamide, MDC] (Sigma-Aldrich, Missouri, USA), into *N*,*N*-dimethyl casein according to the fluorescence method reported (Liu et al. 2014). Twenty microliters of a 12×*Sm*TG solution extracted from either the BY4741-TG or GCI-TG strain was mixed with 460 µL of the mixture (50 mM Tris–HCl (pH 8.0), 0.2% *N*,*N*-dimethyl casein, 13 µM MDC). The assay mixture was incubated for 30 min at 37 °C and the reaction terminated by adding (NH_4_)_2_SO_4_ to a final concentration of 42 mM. The fluorescence intensity was measured by a microplate microplate reader Synergy HTX (BioTek, Vermont, USA) using excitation and emission wavelengths of 350 and 500 nm, respectively.

### Analytical methods

The cell concentration was determined by measuring the OD_600_ using a Gene Quant1300 spectrometer (GE Healthcare Life Sciences, Buckinghamshire, UK). The glutathione concentration was measured via a 5,5′-dithiobis (2-nitorobenzonic acid)-glutathione reductase recycling assay, as described previously (Hara et al. 2009).

## Results

### Heterologous expression of *Sm*TG in *S. cerevisiae*

**Fig. 1:**
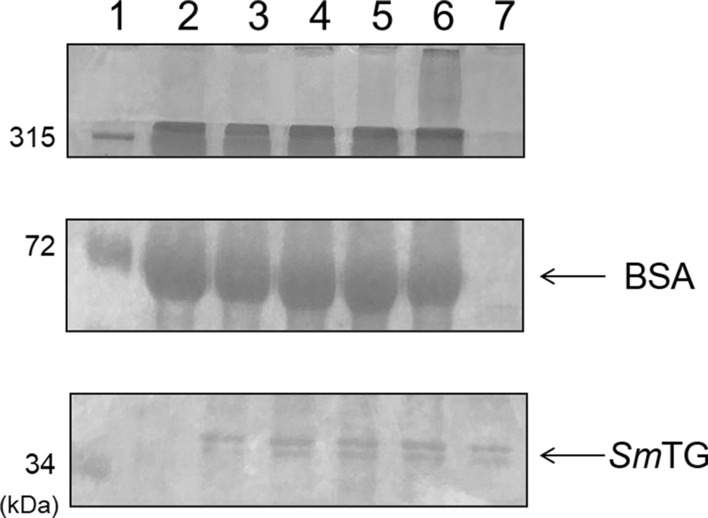
Cross-linking assay using BSA as a substrate after incubation with a *Sm*TG solution extracted from the BY4741-TG strain for several reaction times. Lane 1, molecular size marker; lane 2, BSA; lanes 3, 4, 5 and 6, 1×*Sm*TG (+BSA) for 0, 30, 60 and 120 min, respectively; lane 7, 1×*Sm*TG (−BSA)

The gene encoding *Sm*TG was codon optimized for expression in *S. cerevisiae* and transformed into the *S. cerevisiae* BY4741 strain. Cell growth of BY4741 expressing *Sm*TG (BY4741-TG strain) was similar to that of the control strain. A solution of *Sm*TG was obtained by disrupting the BY4741-TG strain expressing *Sm*TG after cultivation for 24 h. Intracellular expression of *Sm*TG was confirmed by SDS-PAGE analysis by the presence of a band of ~ 37 kDa (Fig. 1). Cross-linking activity of the *Sm*TG solution extracted from the BY4741*-*TG strain was also confirmed using BSA as a substrate. A smeared band was observed when incubating BSA with *Sm*TG, which represented polymerized BSA and the intensity of this smeared band was proportional to the reaction time (Fig. 1). Moreover, more BSA polymerized as the concentration of *Sm*TG was increased (Additional file 1: Fig. S1).

### Effect of glutathione addition on cross-linking activity of *Sm*TG

**Fig. 2:**
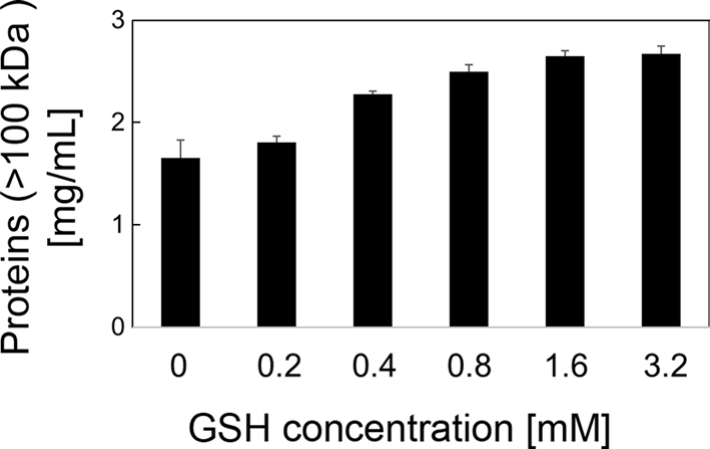
Effect of reduced glutathione (GSH) addition on polymerization of caseinate by *Sm*TG. The concentration of proteins whose molecular weights were more than 100 kDa were detected after polymerization of caseinate by 0.2×*Sm*TG extracted from the BY4741-TG strain in the presence of different GSH concentrations

Glutathione was added to the *Sm*TG solution extracted from the BY4741-TG strain to investigate the affect of glutathione on cross-linking activation of *Sm*TG (Fig. 2 and Additional file 1: Fig. S2). In general, milk proteins are good substrates for *Sm*TG. In particular, caseins, major milk proteins, are a favorable substrate for *Sm*TG to cross-link because they are non-globular and have limited tertiary structure, which makes them readily accessible to enzymes. Among them, both α-casein and κ-casein have two cysteines and have been reported to play an important role in the formation of cross-links (Fox et al. 1998). Proteins that have molecular weights less than 100 kDa were removed by ultrafiltration, and the remaining proteins were quantified to determine the level of caseinate cross-linking by *Sm*TG. As shown in Fig. 2, proteins with molecular weights above 100 kDa increased as the glutathione concentration increased from 0.2 to 0.8 mM (Fig. 2). The concentration of proteins with molecular weights greater than 100 kDa in the presence of 0.8 mM glutathione was 1.5-fold higher when compared with the results obtained in the absence of glutathione. Polymerization was saturated at concentrations above 0.8 mM glutathione (Fig. 2).

### Heterologous overexpression of *Sm*TG in a* S. cerevisiae* strain producing high amounts of glutathione

**Fig. 3:**
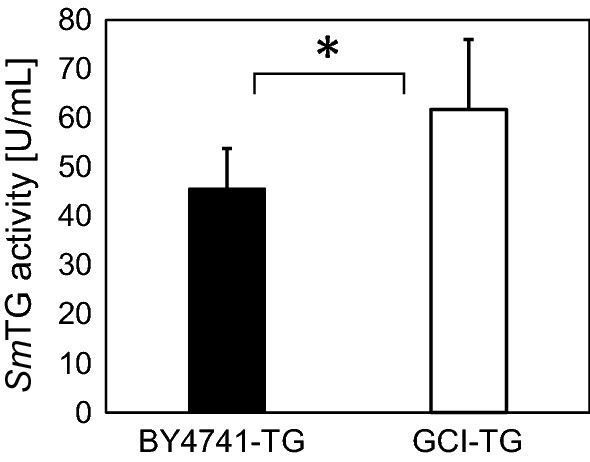
*Sm*TG activity measured by the incorporation of MDC into *N*,*N*-dimethylcasein. 12×*Sm*TG extracted from BY4741-TG and GCI-TG strains were used for cross-linking reactions. The values are means and the error bars show the SD (*n* = 5). Statistically significant differences are indicated by an asterisk (*p* = 0.023)

We previously constructed the *S. cerevisiae* GCI strain, which produces higher levels of intracellular glutathione when compared with the parental *S. cerevisiae* strain (Hara et al. 2012). From the viewpoint of enhancing *Sm*TG activity, *S. cerevisiae* host strain producing enhanced levels of glutathione for expression of *Sm*TG is an attractive approach to enhance *Sm*TG activity. Therefore, in this study, the gene encoding *Sm*TG was overexpressed in the GCI strain. Cell growth and glutathione production were compared between the GCI and BY4741 strains when overexpression of *Sm*TG was induced (Additional file 1: Fig. S3). The cell growth of both strains was similar (Additional file 1: Fig. S3a). In contrast, glutathione content increased drastically in the GCI-TG strain when compared with that of the BY4741-TG strain (Additional file 1: Fig. S3b). The BY4741-TG strain and GCI-TG strain produced 0.0017 mM and 0.16 mM glutathione, respectively, after cultivation for 24 h, although the expression levels of *Sm*TG were the same between the both strains (Additional file 1: Fig. S3c). As shown in Fig. 2, addition of 1.6–3.2 mM glutathione showed saturated activity enhancement. Therefore, the 12×*Sm*TG solution extracted from the GCI-TG strain contatined saturated 1.92 mM glutathione. The 12×*Sm*TG solution extracted from the BY4741-TG strain containing less than 0.2 mM was used as an almost non-enhancement control (Fig. 2). The enhancement of *Sm*TG activity extracted from the BY4741-TG strain and GCI-TG strain were compared (Fig. 3). The enhanced activity of *Sm*TG extracted from the GCI-TG strain was 1.4-fold higher when compared with that of the BY4741-TG strain. This result indicates that activity of *Sm*TG expressed in the GCI strain was enhanced because of the higher glutathione content present when compared with that of the BY4741 strain.

**Fig. 4:**
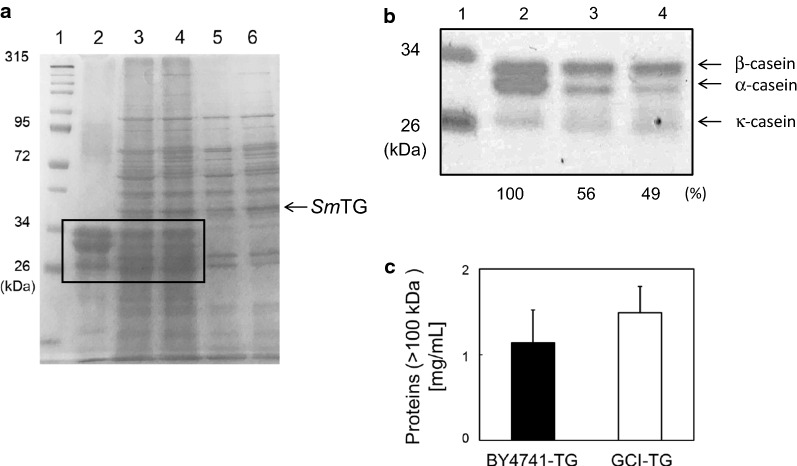
Polymerization of caseinate by *Sm*TG extracted from BY4741-TG and GCI-TG strains. Each sample volume loaded was 20 µL (**a**) and 5 µL (**b**). **a** Lane 1, protein molecular size marker; lane 2, caseinate control; lane 3, polymerized caseins by 12×*Sm*TG extracted from the BY4741-TG strain; lane 4, polymerized caseins by 12×*Sm*TG extracted from the GCI-TG strain; lane 5, BY4741-TG extract; lane 6, GCI-TG extract. **b** Expanded region of the SDS-PAGE in (**a** boxed region). Ratios of cross-linked α-casein were calculated from the intensity of each α-casein band and represented as relative values (%). **c** The concentration of proteins whose molecular weights were more than 100 kDa, were detected after polymerization of caseinate by 12×*Sm*TG extracted from the BY4741-TG and GCI-TG strain

Finally, the cross-linking activity of *Sm*TG extracted from that GCI-TG strain was evaluated using caseinate as a substrate. As shown in Fig. 4a, the intensities of higher molecular-weight bands increased when using the *Sm*TG solution extracted from the GCI-TG strain rather than that from the BY4741-TG strain. The yields of polymerized α-caseins were estimated to be 44% and 51%, because the residual α-caseins were calculated to be 56% and 49% from the BY4741-TG strain and GCI-TG strain, respectively. These results indicate that *Sm*TG extracted from the GCI-TG strain showed enhanced cross-linking activity of α-casein because of the higher content of glutathione present in the GCI-TG strain when compared with that of the BY4741-TG strain. Proteins that have molecular weights less than 100 kDa were removed by ultrafiltration, and the remaining proteins were quantified to determine the level of caseinate cross-linking by *Sm*TG extracted from the BY4741-TG strain and the GCI-TG strain. As shown in Fig. 4c, the concentration of proteins with molecular weights greater than 100 kDa obtained from the GCI-TG strain was 1.3-fold higher when compared with the result obtained from the BY4741-TG strain.

## Discussion

TG is used frequently as an industrial enzyme, especially in the food industry, and reports describing its use in the food industry are numerous (Miwa 2020). The biggest advantage of TG from *S. mobaraense* (*Sm*TG) is its suitability in a variety of applications. In this study, *Sm*TG expressed in *S. cerevisiae* was confirmed to have polymerizing activity. Furthermore, *Sm*TG extracted from the higher glutathione producing *S. cerevisiae*, which overexpresses *GSH*1 and *GSH*2 together with *Sm*TG, also showed activity. The cell growth of both BY4741-TG and GCI-TG strains decreased after 24 h cultivation (Additional file 1: Fig. S3a). This decrease in cell growth is hypothesized to be cell damage caused by polymerization of various intracellular proteins required for survival. However, the reduction in growth rate was small and may not pose as an issue when producing *Sm*TG in *S. cerevisiae*. Polymerization activity of *Sm*TG increased in the presence of glutathione concentrations up to 1.6 mM when caseinate was used as the substrate (Fig. 2). The cross-linking assay was carried out using 12×*Sm*TG extracted from the GCI-TG strain, which contained 1.92 mM glutathione after cultivation for 24 h. The crosslinking activity using 12×*Sm*TG extracted from the GCI-TG strain was enhanced by 1.4-fold when compared with the 12×*Sm*TG extracted from the BY4741-TG strain (Fig. 3). This fold increase is similar to the value (1.5-fold) observed when glutathione was added externally at concentrations above 0.8 mM (Fig. 2). Thus, the production of active *Sm*TG because of the higher levels glutathione produced in the engineered *S. cerevisiae* is advantageous for cross-linking activity by *Sm*TG.

Miwa (2020) reported that crosslinking of pasteurized milk was enhanced in the presence of 0.02–0.2 mM glutathione (Miwa 2020). Mixtures of *Sm*TG and glutathione were supplied from food companies (Bönisch et al. 2007). In the current study, we showed the potential of obtaining a mixture of *Sm*TG and glutathione produced simultaneously by overexpression of *Sm*TG in an engineered *S. cerevisiae* strain producing high levels of glutathione.

Glutathione is industrially produced by fermentation of *S. cerevisiae*. In this study, we succeeded in integrating the production of *Sm*TG and glutathione using *S. cerevisiae* as a host strain. This simple strategy for production of active *Sm*TG in *S. cerevisiae* should reduce production costs and waste as both TG and GSH will be available from one source.

## Supplementary Information

**Additional file 1: Figure S1.** Cross-linking assay using BSA as a substrate after incubation for 120 min with several volumes of *Sm*TG solution extracted from the BY4741-TG strain. Lane 1, molecular size marker; lane 2, BSA only; lanes 3, 4 and 5, 1×, 5× and 10×*Sm*TG (+BSA), respectively; lanes 6, 7 and 8, 1×, 5× and 10×*Sm*TG (−BSA), respectively. **Figure S2.** Effect of adding reduced glutathione to polymerization of caseinate by *Sm*TG. SDS-PAGE profiles show the protein pattern of caseinate after incubation with *Sm*TG. 1×*Sm*TG extracted from the BY4741-TG strain was used. Lane 1, molecular size marker; lane 2, caseinate only; lanes 3, 4, 5, 6, 7 and 8, caseinate, *Sm*TG and 0, 0.2, 0.4, 0.8, 1.6 and 3.2 mM glutathione, respectively; lane 9, *Sm*TG only. The polymerized caseins (molecular weight > 100 kDa) presented in the top panel were recovered and measured as shown in Fig. 2. **Figure S3.** Growth and glutathione production of *S. cerevisiae *expressing *Sm*TG. (a) Time course of cell growth. Solid and dotted lines represent the BY4741-TG and GCI-TG strains, respectively. (b) Glutathione concentrations in 1×*Sm*TG solutions extracted from the BY4741-TG strain (black) and GCI-TG strain (white). (c) Expression level of *Sm*TG after cultivation for 24 h. lane 1, BY4741-TG strain; lane 2, GCI-TG strain. Expression level of *Sm*TG was calculated from the intensity of each *Sm*TG band and represented as relative values (%). The values are means and the error bars show the SD (*n* = 3).

## Data Availability

All data and materials are available.
