# Supplementary material for: Production of transglutaminase in glutathione-producing recombinant Saccharomyces cerevisiae
Source: AMB Express. 2021 Jan 7;11:13. doi: 10.1186/s13568-020-01176-3 (PMC7790930; doi:10.1186/s13568-020-01176-3)
Supplement: Supplementary file 1 — Additional file 1: Figure S1. Cross-linking assay using BSA as a substrate after incubation for 120 min with several volumes of SmTG solution extracted from the BY4741-TG strain. Lane 1, molecular size marker; lane 2, BSA only; lanes 3, 4 and 5, 1×, 5× and 10×SmTG (+BSA), respectively; lanes 6, 7 and 8, 1×, 5× and 10×SmTG (−BSA), respectively. Figure S2. Effect of adding reduced glutathione to polymerization of caseinate by SmTG. SDS-PAGE profiles show the protein pattern of caseinate after incubation with SmTG. 1×SmTG extracted from the BY4741-TG strain was used. Lane 1, molecular size marker; lane 2, caseinate only; lanes 3, 4, 5, 6, 7 and 8, caseinate, SmTG and 0, 0.2, 0.4, 0.8, 1.6 and 3.2 mM glutathione, respectively; lane 9, SmTG only. The polymerized caseins (molecular weight > 100 kDa) presented in the top panel were recovered and measured as shown in Fig. 2. Figure S3. Growth and glutathione production of S. cerevisiae expressing SmTG. (a) Time course of cell growth. Solid and dotted lines represent the BY4741-TG and GCI-TG strains, respectively. (b) Glutathione concentrations in 1×SmTG solutions extracted from the BY4741-TG strain (black) and GCI-TG strain (white). (c) Expression level of SmTG after cultivation for 24 h. lane 1, BY4741-TG strain; lane 2, GCI-TG strain. Expression level of SmTG was calculated from the intensity of each SmTG band and represented as relative values (%). The values are means and the error bars show the SD (n = 3). [file 13568_2020_1176_MOESM1_ESM.docx]

Supplementary Data

*AMB Express*

Production of transglutaminase in glutathione-producing recombinant *Saccharomyces cerevisiae*

Yoko Hirono-Hara^1^, Miyuu Yui^1^, Kiyotaka Y. Hara^1,2,*^

[y-hirono@u-shizuoka-ken.ac.jp](mailto:y-hirono@u-shizuoka-ken.ac.jp)

[miyuu2018@icloud.com](mailto:miyuu2018@icloud.com)

[k-hara@u-shizuoka-ken.ac.jp](mailto:k-hara@u-shizuoka-ken.ac.jp)

^1^Department of Environmental and Life Sciences, School of Food and Nutritional Sciences, University of Shizuoka, 52-1 Yada, Suruga-ku, Shizuoka 422-8526, Japan

^2^Graduate Division of Nutritional and Environmental Sciences, University of Shizuoka, 52-1 Yada, Suruga-ku, Shizuoka 422-8526, Japan

Yoko Hirono-Hara and Miyuu Yui contributed equally to this work.

*Correspondence: Kiyotaka Y. Hara, Graduate Division of Nutritional and Environmental Sciences, University of Shizuoka, 52-1 Yada, Suruga-ku, Shizuoka 422-8526, Japan

E-mail: [k-hara@u-shizuoka-ken.ac.jp](mailto:k-hara@u-shizuoka-ken.ac.jp)

Tel/fax: +81-54-264-5659

**Fig. S1** Cross-linking assay using BSA as a substrate after incubation for 120 min with several volumes of *Sm*TG solution extracted from the BY4741-TG strain. Lane 1, molecular size marker; lane 2, BSA only; lanes 3, 4 and 5, 1×, 5× and 10×*Sm*TG (+BSA), respectively; lanes 6, 7 and 8, 1×, 5× and 10×*Sm*TG (−BSA), respectively


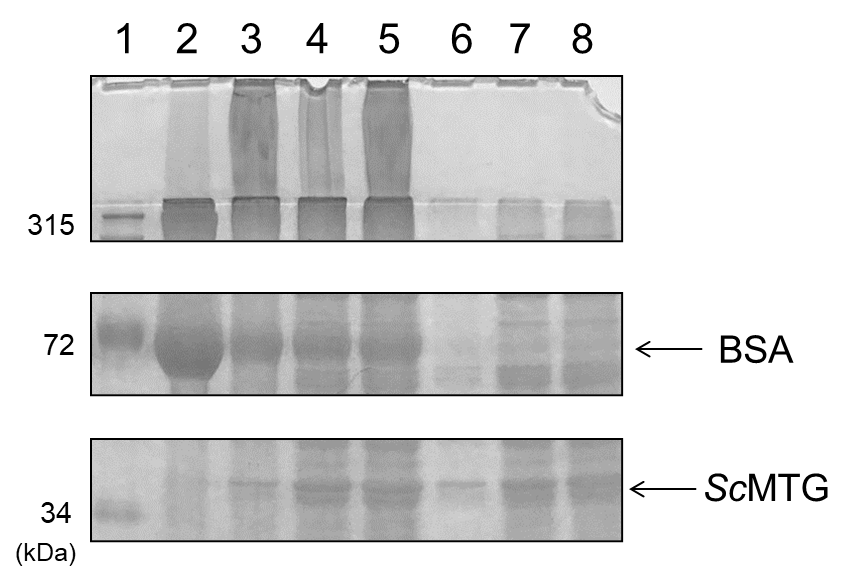


*Sm*TG

BSA


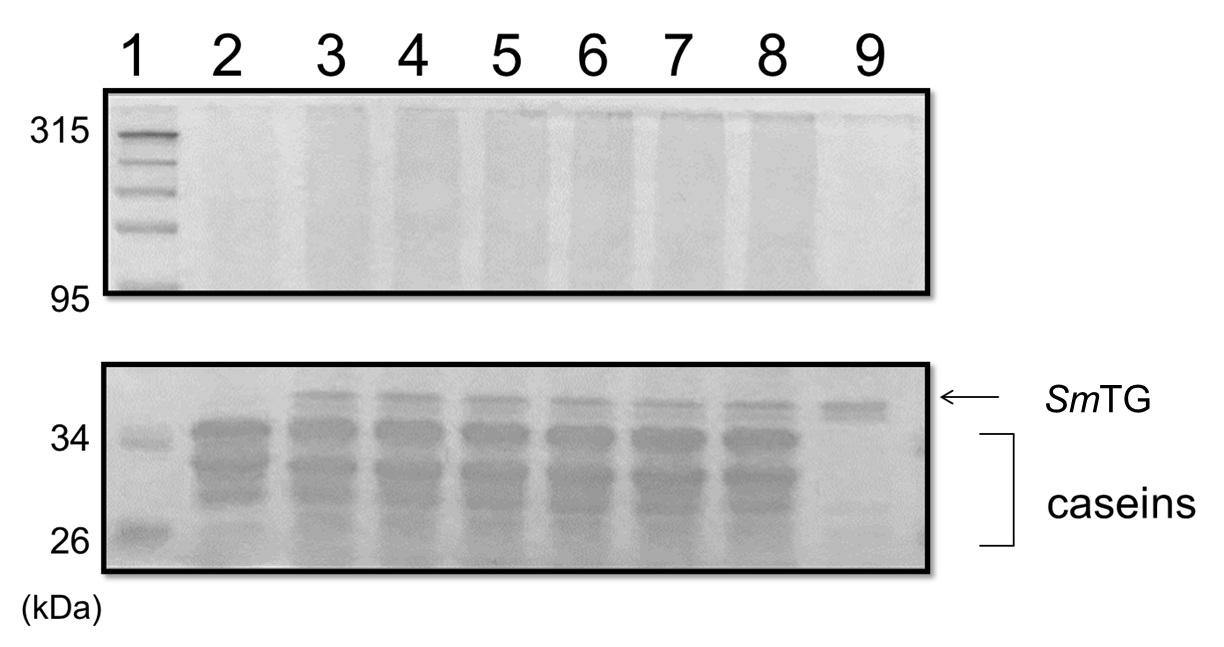


**Fig. S2** Effect of adding reduced glutathione to polymerization of caseinate by *Sm*TG. SDS-PAGE profiles show the protein pattern of caseinate after incubation with *Sm*TG. 1×*Sm*TG extracted from the BY4741-TG strain was used. Lane 1, molecular size marker; lane 2, caseinate only; lanes 3, 4, 5, 6, 7 and 8, caseinate, *Sm*TG and 0, 0.2, 0.4, 0.8, 1.6 and 3.2 mM glutathione, respectively; lane 9, *Sm*TG only. The polymerized caseins (molecular weight > 100 kDa) presented in the top panel were recovered and measured as shown in Fig. 2


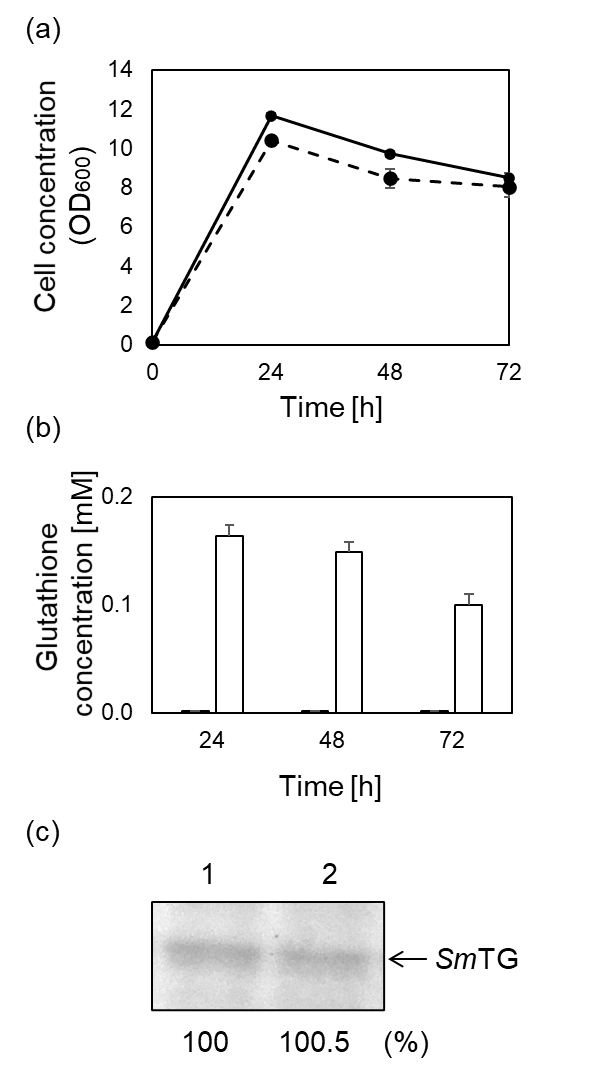


**Fig. S3** Growth and glutathione production of *S. cerevisiae* expressing *Sm*TG. (a) Time course of cell growth. Solid and dotted lines represent the BY4741-TG and GCI-TG strains, respectively. (b) Glutathione concentrations in 1×*Sm*TG solutions extracted from the BY4741-TG strain (black) and GCI-TG strain (white). (c) Expression level of *Sm*TG after cultivation for 24 h. lane 1, BY4741-TG strain; lane 2, GCI-TG strain. Expression level of *Sm*TG was calculated from the intensity of each *Sm*TG band and represented as relative values (%). The values are means and the error bars show the SD (*n* = 3)
